# Seasonal Changes in the Structure and Function of Gut Microbiota in the Muskrat (*Ondatra zibethicus*)

**DOI:** 10.3390/metabo13020248

**Published:** 2023-02-09

**Authors:** Fengcheng Song, Yishu Xu, Peng Peng, Hongxu Li, Ranxi Zheng, Haolin Zhang, Yingying Han, Qiang Weng, Zhengrong Yuan

**Affiliations:** College of Biological Sciences and Technology, Beijing Forestry University, Beijing 100083, China

**Keywords:** gut microbiota, metabolism, muskrat, seasonal breeding, metagenome

## Abstract

The gut microbiota plays a crucial role in the nutrition, metabolism, and immune function of the host animal. The muskrat (*Ondatra zibethicus*) is a typical seasonal breeding animal. The present study performed a metagenomic analysis of cecum contents from muskrats in the breeding and non-breeding seasons. The results indicated that the breeding muskrats and non-breeding muskrats differed in gut microbiota structure and function. During the breeding season, the relative abundance of phylum Bacteroidetes, genus *Prevotella*, and genus *Alistipes* increased, while the relative abundance of phylum Firmicutes and phylum Actinobacteria decreased. The muskrat gut microbiota was enriched in the metabolism-related pathways, especially amino acid and vitamin metabolism, and genetically related metabolites in the breeding season. We presumed that the muskrat gut microbiota might seasonally change to secure reproductive activity and satisfy the metabolic demands of different seasons. This study could explore potential mechanisms by which gut microbiota affects reproduction. Moreover, this study may provide a new theoretical basis for the management of muskrat captive breeding.

## 1. Introduction

The gut microbiota refers to the diverse microorganisms present in the digestive system of animals, which plays a vital role in animal metabolism, immunity, and reproduction [1,2,3]. Sometimes it is called a “forgotten organ” [4]. For the past few years, depending on the rapid development of bioinformatics, especially the rise of metagenomics, the functions of gut microbiota are being gradually understood [5,6]. The most important function of the intestinal microbiota is the nutritional function, providing energy to the host. Up to 35% of the digestive and metabolic enzymes in mammals are secreted by gut microbiota [7]. The gut microbiota is not static. It is diverse and unstable and is highly susceptible to external environmental influences, such as food [8,9], age [10], disease [11], and living areas [12,13]. The gut microbiota of the same species can vary greatly at different times and in different environments, which can help the host to adapt to its surroundings by influencing host energy metabolism or other aspects.

Recently, numerous studies have revealed that animal reproduction is closely linked to gut microbiota. *Clostridium scindens* American Type Culture Collection 35,704 can convert glucocorticoids to androgens via side-chain cleavage [14]. The gut microbiota can also be involved in gut metabolism and deglucuronidation of dihydrotestosterone (DHT) and testosterone (T) and results in higher DHT levels in the colon of young healthy mice than in germ-free mice [15]. Accordingly, studies for the composition and function of the gut microbiota might be essential for further research on animal reproduction.

Seasonal breeding is a phenomenon in which some animals mate only at certain times of the year. Seasonal breeding activity may be influenced mainly by photoperiodism [16]. Photoperiodic changes are sensed by the pineal gland in the brain. It secretes melatonin at night to regulate the secretion of the Gonadotropin-releasing hormone and the Luteinizing hormone, leading to seasonal changes in reproductive activity [17]. Meanwhile, several studies have confirmed that many other factors, such as estrogen, thyroid hormone, kisspeptin, and living environment, affect seasonal breeding animals [18,19,20,21]. According to a recent study, the gut microbiota may regulate seasonal breeding and behavior based on photoperiodic timing in rodents via the Hypothalamic Pituitary Gonadal (HPG) axis, melatonin, and the Kisspeptin/G-protein coupled receptor 54 (GPR54) system in the hypothalamus [22]. This report has aroused our interest in the further investigation of the association between gut microbiota and seasonal breeding.

The muskrat (*Ondatra zibethicus*) is a seasonal breeding animal. It has a breeding season from March to October and a non-breeding season from November to February [23]. During the breeding season, muskrats secrete a substance called musk, which is highly valued for its medicinal properties [24,25]. Most of the previous studies on muskrats have focused on the differences between the reproductive organs or secretory glands of muskrats during the breeding and non-breeding seasons [23,26,27]. Nevertheless, the variation in the composition and function of the gut microbiota of muskrats in different seasons is unknown, and the correlation between gut microbiota and seasonal reproductive phenomena in muskrats has not been explored.

The present study utilized high-throughput sequencing by metagenome sequencing to analyze the variation in the structure and function of the muskrat gut microbiota during different seasons. α and β diversity analysis, LEfSe analysis, Metastats analysis, and functional difference analysis on sequencing results were performed to explain differences in gut microbiota. This study aimed to investigate the seasonal variation of composition and function in the gut microbiota of muskrats, further study the secrets of seasonal breeding in muskrats and provide a new theoretical basis for the progress of captive breeding of muskrats.

## 2. Materials and Methods

### 2.1. Sample Collection

Adult male muskrats were obtained in January 2022 (the non-breeding season, *N* = 5) and May 2022 (the breeding season, *N* = 5) from a muskrat breeding base in Xinji, Hebei, China. Based on the references given by the breeding base, the nutritional compositions of the diets of muskrats for each season are as follows. Breeding season: total energy 19.84 MJ/kg, dry matter 36 g, crude protein 7.2 g, crude fiber 5.4 g, and crude fat 1.4 g. Non-breeding season: total energy 15.95 MJ/kg, dry matter 30 g, crude protein 3.6 g, crude fiber 9.0 g, and crude fat 1.1 g. Additionally, vitamins A, D, and E were added to the diet during the breeding season for better reproduction of muskrats [28,29]. Animals were executed after being anesthetized (CO_2_ inhalation) [30], and the animals were dissected using antiseptic equipment to collect cecum contents. As soon as the cecum contents were collected, they were frozen in liquid nitrogen. All animal experimental procedures were approved by the Institutional Review Board (or Ethics Committee) of Beijing Forestry University (protocol code EAWC_BJFU_2021004).

### 2.2. Metagenomic Sequencing and Data Processing

Using the TIANamp Stool DNA Kit (TIANGEN BIOTECH, Beijing, China), the total genomic DNA was extracted from cecum contents. The libraries were constructed through the Enzymic Universal DNAseq Library Prep Kit (Kaitai-Bio, Zhejiang, China), and then the libraries were tested for quality control. The qualifying libraries were sequenced on the Illumina NovaSeq PE150 platforms (Illumina, San Diego, CA, USA). To obtain clean data for further analysis, the raw data were pre-processed to remove low-quality and host sequence contamination. The clean metagenome data assembly was performed using Megahit 1.2.9 [31]. In the gene assembly, we keep the sequences (contigs) with lengths longer than 500 bp [32,33,34] for subsequent analysis. Gene prediction was performed on contigs using MetaGeneMark v.3.38 (Georgia Institute of Technology, Atlanta, GA, USA) [35,36,37], clustering with identity 95%, converge 90% [38,39], and statistical Unigene abundance information by Salmon 1.8.0 [40].

### 2.3. Analysis of Species Composition and Function

Gene prediction information was compared with the Non-Redundant Protein Sequence Database (NR), evolutionary genealogy of genes: Non-supervised Orthologous (eggNOG) database [41,42] and Kyoto Encyclopedia of Genes and Genomes (KEGG) PATHWAY database [43,44] using DIAMOND 2.0.7 (Max Planck Institute for Biology, Tübingen, Germany)(blastp, evalue ≤ 1 × 10^−5^) [45,46]. Species-specific taxonomic information at all levels (phylum, class, order, family, genus, and species) was obtained by MEGAN 6.21.2 (Tübingen University, Tübingen, Germany) [47,48]. The Bray–Curtis distance used for β-diversity was calculated and visualized with principal coordinate analysis (PCoA). Biomarkers with significant variation between groups were ascertained using linear discriminant analysis effect size (LEfSe) [49]. The images were drawn through R 4.1.1 or the online site ImageGP [50]. Analysis of functional pathways with significant differences was conducted using STAMP 2.1.3 [51] and the Metastats method [52].

## 3. Results

### 3.1. Metagenomic Sequencing and Gene Prediction

We performed sequencing on muskrat cecum contents samples and obtained a total of 117.50 gigabases (Gbps) of raw data. The raw data were quality controlled to obtain 115.52 Gbps of clean data. The quality control efficiency of the clean data was 98.29% (Table S1). A total of 1,944,649 contigs with the longest length of 585,984 bp were obtained (Table S2).

**Figure 1:**
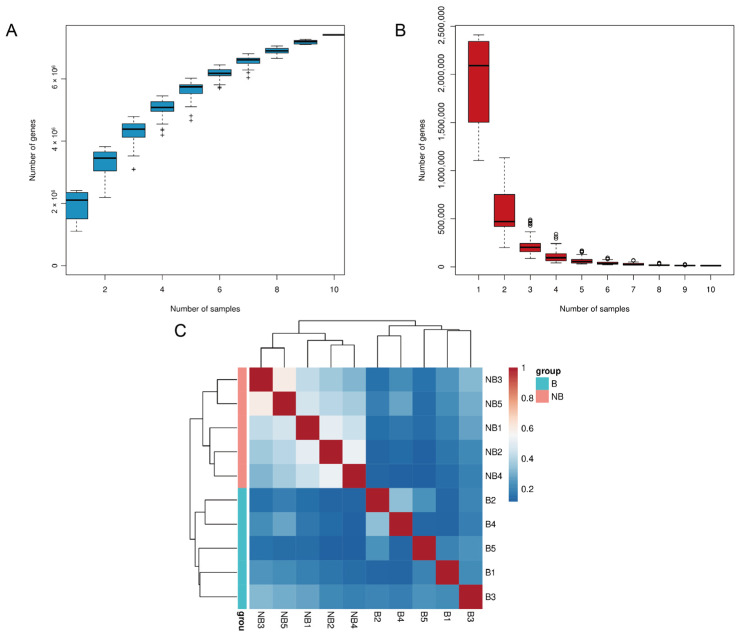
Statistical analysis of gene information. The rarefaction curve for pan gene (**A**) and core gene (**B**) (“+” and “○” represent outliers). (**C**) The heat map of correlation among samples.

A total of 7,414,843 open reading frames and a total length of 4411.7 Mbp Unigene were obtained for gene prediction (Table S3). The gene information was used to construct a core-pan gene rarefaction curve to evaluate the sample sequencing depth (Figure 1A,B). The curve tends to increase and flatten out with increasing sample size, proving that our sequencing results have largely covered all species. The sample size was reasonable for our study. Meanwhile, the correlation heat map was constructed, and the richness varied greatly between groups, which further proved the reliability of the experiment (Figure 1C).

### 3.2. Gut Microbiota Composition and Difference

**Figure 2:**
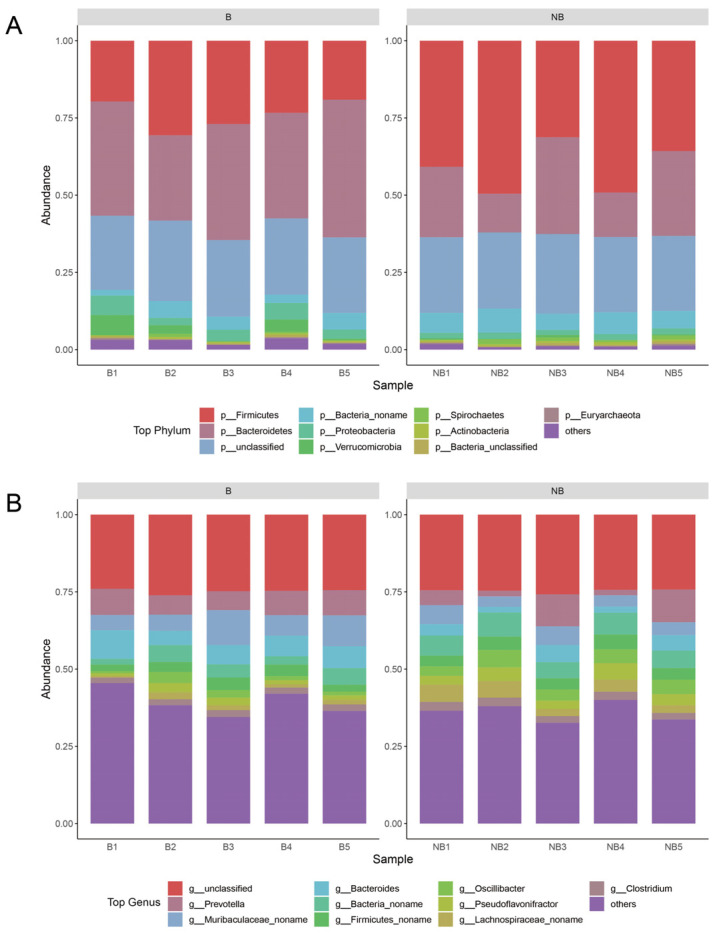
Species composition between the breeding (B) and non-breeding seasons (NB). The top ten species in relative abundance for each taxon were selected for plotting, and the remaining species were categorized as others. (**A**) The stack bar plot of phylum-level species composition. (**B**) The stack bar plot of genus-level species composition.

The following analyses were performed using the abundance information obtained from the species annotations. The analysis of the top 10 microbiota in relative abundance at the phylum level and genus level were plotted separately (Figure 2). At the phylum level, the dominant phylum was Bacteroidetes and Firmicutes in both the breeding and non-breeding seasons, and the mean abundance of Firmicutes was higher in the non-breeding season (41.30%) than in the breeding season (23.94%), while the mean abundance of Bacteroidetes was lower (21.71%) than in the breeding season (36.19%). From the genus perspective, only 24.76% of sequences were unclassified. *Prevotella* (6.63%), Muribaculaceae_noname (6.15%), *Bacteroides* (5.20%), and Bacteria_noname (5.19%) had high relative abundance (>5%) during both the breeding and non-breeding seasons. The detailed abundance table was shown in Table S4.

**Figure 3:**
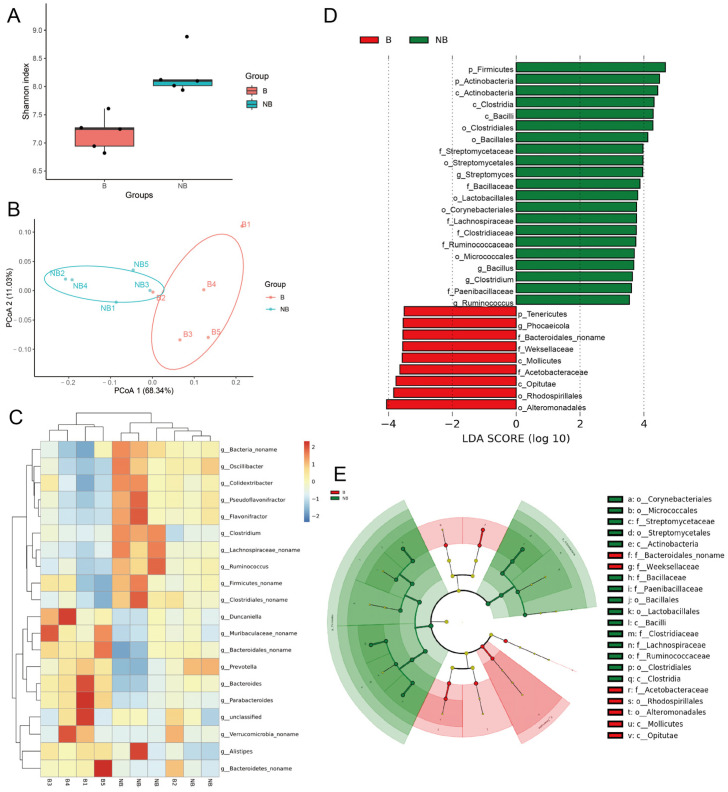
Bioinformatics analysis of the differences in gut microbiota composition between the breeding (B) and non-breeding (NB) seasons. (**A**) The Shannon’s index of α-diversity analysis (*p* < 0.05). (**B**) The Principal Coordinate Analysis (PCoA) is based on the Bray–Curtis distance with significant differences in genus levels between the two groups. (**C**) The genus-level abundance heat map shows the top 20 genera in relative abundance. (**D**,**E**) The LEfSe plots show taxa differing between groups. (**D**) The bar plot shows the distribution of LDA values for the differential biomarkers, and the histogram of LDA value distribution shows the species with significant differences (LDAScore > 4). (**E**) The cladogram shows the evolutionary branching of divergent species.

α-Diversity analysis showed significantly higher gut microbiota richness in the non-breeding season muskrats than in the breeding season (Figure 3A). PCoA at the genus level can be used to reveal the variation in the gut microbiota of muskrats between the breeding and non-breeding seasons (Figure 3B). Samples from different seasons were well clustered together, with PCo1 explaining 68.34% of the total variation in samples and PCo2 explaining 11.03% of the total variation in samples, indicating that the gut microbiota of muskrats varied at the genus level in different seasons. *Prevotella*, *Bacteroides*, and *Alistipes* were considerably higher in the breeding season than in the non-breeding season, whilst *Flavonifractor*, *Oscillibacter*, and *Colidextribacter* were significantly higher in the non-breeding season than in the breeding season (Figure 3C). LEfSe demonstrated significantly different biomarkers between groups (Figure 3D,E). The non-breeding season microbiota substantially enriched in phylum Firmicutes and phylum Actinobacteria. In contrast, phylum Tenericutes and order family Acetobacteraceae enriched in the breeding season. These results further validated the difference in gut microbiota between the breeding season and the non-breeding season.

### 3.3. Functional Analysis of Gut Microbiota in Muskrat

**Figure 4:**
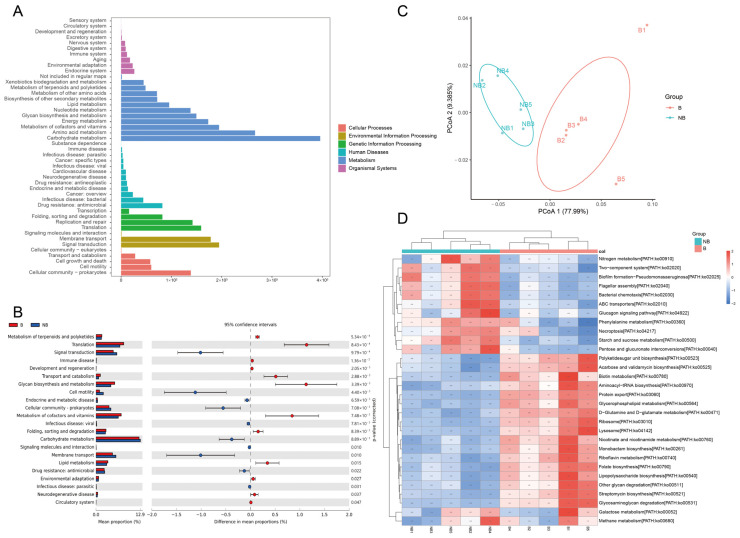
Functional prediction analysis of the muskrat gut microbiota based on the KEGG database. (**A**) The statistical plot of KEGG annotations of the muskrat gut microbiota. (**B**) The top 20 significant functional difference analyses of muskrat gut microbiota in the KEGG level 2 pathway (*p* < 0.05). (**C**) The functional prediction of muskrat gut microbiota in KEGG pathway by Principal coordinate analysis (PCoA). (**D**) The heat map of significantly different top 30 KEGG pathways (*p* < 0.01).

**Figure 5:**
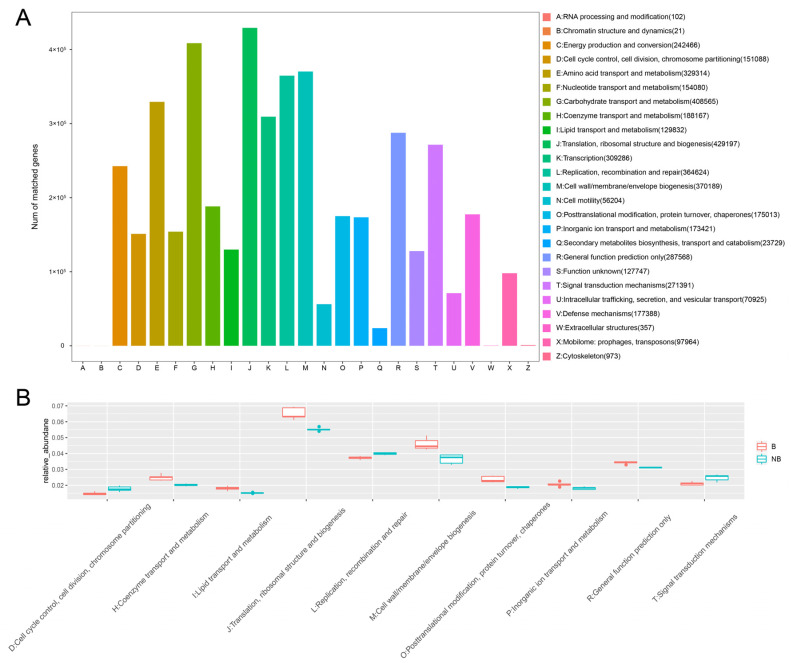
Functional prediction analysis of the muskrat gut microbiota based on the eggNOG database. (**A**) The bar plot of eggNOG annotations of the muskrat gut microbiota. (**B**) The box plot based on Metastats analysis data shows the top 10 functions with significant differences.

To investigate the metabolic-related changes in the gut microbiota during the breeding and non-breeding seasons, the KEGG pathway (Figure 4A) and eggNOG (Figure 5A) analyses were performed using DIAMOND. KEGG pathways were mainly enriched in metabolism (Figure 4A), including carbohydrate metabolism (12.65%), amino acid metabolism (9.26%), metabolism of cofactors and vitamins (6.90%), energy metabolism (6.40%), and glycan biosynthesis and metabolism (4.88%). Other pathways were genetic information processing (18.95%), environmental information processing (13.24%), cellular processes (9.66%), human diseases (6.70%), and organismal systems (3.12%) (Table S5). Difference analysis of the KEGG level2 pathways (*p* < 0.05) showed that there are 10 differentially significant pathways enriched in the non-breeding season, such as the carbohydrate metabolism, cell motility, signal transduction, drug resistance: antimicrobial, endocrine and metabolic disease, infectious disease: parasitic, cellular community-prokaryotes, infectious disease: viral, membrane transport, and signaling molecules and interaction. There are 12 differentially significant pathways enriched in the breeding season, such as the circulatory system, development and regeneration, environmental adaptation, folding, sorting and degradation, neurodegenerative disease, immune disease, glycan biosynthesis and metabolism, lipid metabolism, metabolism of cofactors and vitamins, metabolism of terpenoids and polyketides, translation and transport, and catabolism (Figure 4B).

PCoA of specific pathways showed a clear separation between the breeding and non-breeding samples (Figure 4C). The heat map showed the clustering of metabolic pathways with significant differences in relative abundance in the top 30 (*p* < 0.01). The breeding season was enriched with many functional pathways related to the amino acid, cofactor, and vitamin metabolism, such as biotin metabolism, D-Glutamine and D-glutamate metabolism, folate biosynthesis, and nicotinate and nicotinamide metabolism (Figure 4D). The plot of all significantly different KEGG pathway analyses was shown in Figure S1.

The 25 functions of the eggNOG database were annotated (Figure 5A). The major functions (annotated Unigene > 100,000) were translation, ribosome structure, and biogenesis (J), carbohydrate transport and metabolism (G), cell wall/membrane/envelope biogenesis (M), replication, recombination, and repair (L), amino acid transport and metabolism (E), transcription (K), general function prediction only (R), signal transduction mechanism (T), energy production and conversion (C), coenzyme transport and metabolism (H), defense mechanism (V), posttranslational modification, protein turnover, and chaperones (O), inorganic ion transport and metabolism (P), nucleotide transport and metabolism (F), cell cycle control, cell division, and chromosome partitioning (D), lipid transport and metabolism (L), and function unknown (S). The top 10 significantly different functions obtained using the Metastats method were shown in Figure 5B. During the breeding season, several metabolism-related functions were enriched: coenzyme transport and metabolism (H), lipid transport and metabolism (I), and inorganic ion transport and metabolism (P). The functional analysis illustrated that the gut microbiota of breeding season and non-breeding season muskrats differed significantly in the roles that they play in their hosts.

## 4. Discussion

As a typical seasonal breeding animal, changes in the gut microbiota of the muskrat with the seasons are of great curiosity. There are many studies describing the phenomenon of seasonal changes in gut microbiota, such as rats (*Rattus norregicus*) [53], Tibetan macaques (*Macaca thibetana*) [54], and Siberian hamsters (*Phodopus sungorus*) [55]. Seasonal differences were also found in the gut microbiota of forest musk deer (*Moschus* spp.), an animal capable of musk secretion similar to the muskrat [56]. The present study analyzed the changes in the gut microbiota of muskrats during different breeding seasons. The results showed that there was significant variation in the structure and function of the gut microbiota between the breeding and non-breeding muskrats. These results suggested the possible involvement of gut microbes in meeting the various demands of seasonal breeding in muskrats.

Seasonal changes may influence the structure of the muskrats’ gut microbiota. Results of alpha diversity analysis showed that the breeding and non-breeding seasons differed significantly and that the gut microbial richness was higher in the non-breeding season. Previous studies have shown that a higher abundance of gut microbiota helps hosts adapt to the external environment and enhances their resistance to adverse external conditions [57,58]. Therefore, we hypothesized that the increased abundance of gut microbiota in the non-breeding muskrats during winter may enhance resistance to factors such as the cold. The dominant phyla of the gut microbiota of the muskrat were Bacteroidetes and Firmicutes, which was consistent with our previous findings on another rodent seasonal breeder, the wild ground squirrel (*Spermophilus dauricus*) [59]. The dominant phylum of other rodents such as arctic ground squirrels (*Urocitellus parryii*) was also Bacteroidetes and Firmicutes [60]. Bacteroidetes and Firmicutes are widespread in animals and are a major component of healthy gut microbiota [61]. Their main function is to break down carbohydrates in the gut and synthesize short-chain fatty acids (SCFAs) to provide energy to the host [62]. Many studies have shown that changes in the ratio of Bacteroidetes and Firmicutes may be associated with obesity and that an increased *Firmicutes*/*Bacteroidetes* ratio may lead to obesity and other diseases [63,64]. The abundance of Bacteroidetes increased in the breeding season, whilst Firmicutes significantly increased in the non-breeding season. This change may lead to more stable energy metabolism during the breeding season and safeguard the breeding activity of muskrats.

Muskrats in the breeding season have a higher protein requirement, about 7.2 g of protein per 36 g of dry matter, which is greater than in the non-breeding season (winter). The winter diet should not be high in protein and fat to avoid over-fattening the animal [65]. Genus *Prevotella* and genus *Alistipes* were enriched in the breeding season, genus *Oscillibacter*, order Lactobacillales, family Lachnospiraceae, and family Ruminococcaceae were enriched in the non-breeding season. In a previous study, *Prevotella* and *Alistipes* were enriched in Brandt’s voles (*Lasiopodomys brandtii*) with long photoperiods [22], which were reflected in the present study for the breeding season. *Alistipes* were positively correlated with cholesterol metabolism [66], which may be related to the high-fat, high-protein diet structure of breeding muskrats. *Prevotella* had a higher diversity, and it has been suggested that it may promote increased glycogen stores [67], while the study by Chen et al. also demonstrated that *Prevotella* was positively associated with fat accumulation in pigs [68].

Genus *Oscillibacter* has been reported to be positively associated with a reduction in body weight, which is an important reference for weight control in non-breeding muskrats [69]. The enrichment of some bacteria of order Lactobacillales has been found to cause a decrease in sperm viability, which may be the reason why they were enriched in the non-breeding season rather than the breeding season [70]. Additionally, the family Lachnospiraceae, order Lactobacillales, and family Ruminococcaceae can hydrolyze starch and other sugars to produce butyrate and other SCFAs [71,72]. The non-breeding muskrats with increased carbohydrate content in their diet and reduced total food intake are likely to encounter energy-deficient conditions [65], and these gut microorganisms provide effective energy for muskrats over the winter.

The present study also predicted and analyzed the function of the muskrat gut microbiota based on the KEGG and eggNOG databases. The results of the analysis showed that most of the functions of muskrat gut microbiota were enriched in metabolism-related pathways, especially carbohydrate metabolism. Sixty percent of the muskrat’s diet is composed of carbohydrates, which are the main source of energy for the muskrat. Other major pathways involved are amino acid metabolism, metabolism of cofactors and vitamins, energy metabolism, lipid metabolism, etc. There are significant seasonal differences in all these functions. Muskrats, similar to other monogastric animals, can automatically regulate the amount of food that they eat to meet their energy requirements. During the breeding season, the muskrat’s diet contains more animal feed and higher levels of proteins, lipids, and other nutrients, so the metabolism pathways associated with amino acids, lipids, vitamins, and cofactors increase significantly during the breeding season to meet the complex food structure of the muskrat during that season. In contrast, during the non-breeding season, muskrats eat less and have a high proportion of plant-based feeds, while the lower ambient temperature was not conducive to their energy supply, and the enriched carbohydrate metabolic pathways at this time can help muskrats better survive in this period.

This study investigated seasonal differences in the gut microbiota of muskrats using a metagenomic approach. The present study initially explored the correlation between gut microbiota and seasonal breeding and speculated on the possible regulatory mechanisms of gut microbiota on seasonal breeding in muskrats. This research can provide a new theoretical basis for muskrat captive breeding and help breeders understand the seasonal changes in muskrat metabolism. They can use this as a basis to better regulate the diet structure of muskrats in different seasons. Furthermore, more work needs to be conducted in the future to clarify the composition and function of the gut microbes in muskrats and to further explore the important role of the gut microbiota in seasonal breeding animals.

## 5. Conclusions

There were notable seasonal differences in the species composition and structure of the gut microbiota of muskrats. Gut microbiota richness increased in muskrats during the non-breeding season, which may help muskrats to increase their resistance to the external environment. At the phylum level, the relative abundance of *Bacteroidetes* was increased and the relative abundance of *Firmicutes* was decreased in the gut microbiota of breeding muskrats. The genus *Prevotella* and genus *Alistipes* enriched in the breeding season, and the genus *Oscillibacter*, order Lactobacillales, family Lachnospiraceae, and family Ruminococcaceae enriched in the non-breeding season. The muskrat’s gut microbiota was highly enriched in nutrient metabolic pathways. Differential microbiotas during the breeding season were mainly enriched in amino acid, lipid, vitamin, and cofactor metabolism pathways to help muskrats better reproductive activities. This study may provide a new theoretical basis for managing muskrat captive breeding and lay the foundation for further ensuring the efficient breeding of muskrats.

## Data Availability

Data is contained within the article and Supplementary Material.

## Supplementary Materials

The following supporting information can be downloaded at: https://www.mdpi.com/article/10.3390/metabo13020248/s1, Figure S1: Analysis of all *p* < 0.01 differential metabolic pathways. Table S1: Clean data information after quality control of raw data from sequencing. Table S2: Gene assembly result evaluation. Table S3: Gene prediction and abundance statistics. Table S4: Abundance statistics for all taxonomic levels (phylum, order, family, genus, and species). Table S5: Abundance statistics for KEGG pathways.
