# Supplementary figures and images for: Seasonal Changes in the Structure and Function of Gut Microbiota in the Muskrat (Ondatra zibethicus)
Source: Metabolites. 2023 Feb 9;13(2):248. doi: 10.3390/metabo13020248 (PMC9966595; doi:10.3390/metabo13020248)

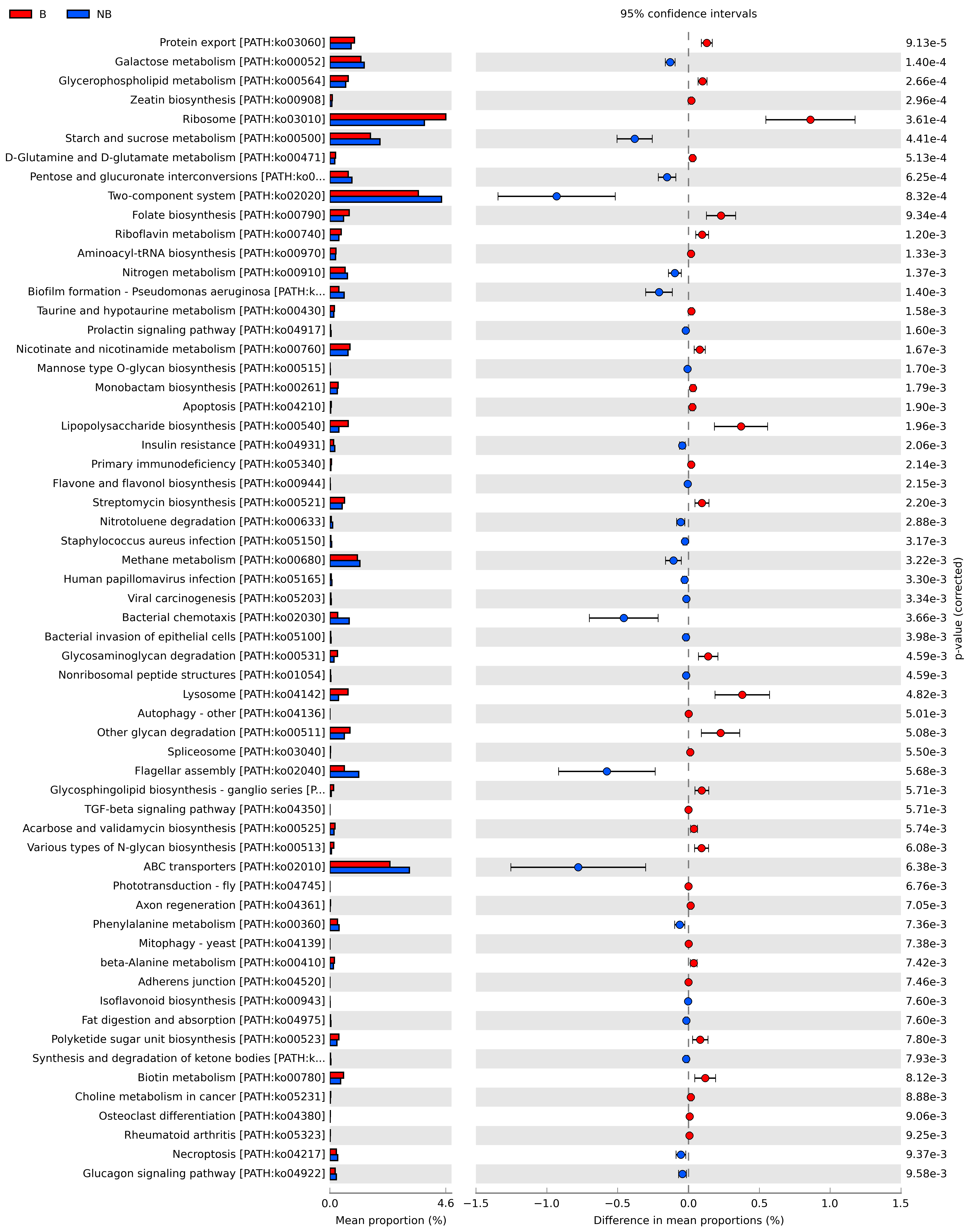

Supplement: Supplementary file 1 [file metabolites-13-00248-s001.zip › Supplementary Materials/Figure S1.png]
